# The accordion worm: a new genus and species of heteronemertean (Nemertea, Pilidiophora) from Galicia (Spain)

**DOI:** 10.1098/rsos.250313

**Published:** 2025-05-07

**Authors:** Aida Verdes, Carlota Gracia-Sancha, Jacinto Pérez-Dieste, María Conejero, Patricia Alvarez Campos, Carlos Leiva, Sergi Taboada, Ana Riesgo, Juan Junoy

**Affiliations:** ^1^ Biodiversity and Evolutionary Biology Department, National Museum of Natural Sciences, Madrid, Spain; ^2^ Grupo de Estudos do Medio Mariño (GEMM), A Coruña, Spain; ^3^ Department of Biology, Universidad Autonoma de Madrid, Madrid, Spain; ^4^ University of Guam, Mangilao, Guam, Guam; ^5^ Department of Life Sciences, Natural History Museum, London, UK; ^6^ Departamento de Ciencias de la Vida, Universidad de Alcalá, Alcala de Henares, Spain

**Keywords:** accordion worm, Heteronemertea, Lineidae, Nemertea, *Pararosa vigarae*, ribbon worm

## Abstract

Ribbon worms (Nemertea) are a less-known group of invertebrates, specially challenging for taxonomic studies due to the scarcity of external morphological features. As a consequence, the number of known nemertean species might represent just a small fraction of the true diversity of the phylum. The present study increases the number of known ribbon worm species with the description of the accordion worm *Pararosa vigarae* sp. nov., a new genus and species of Heternonemertea from the northwest coast of Spain. We performed molecular phylogenetic analyses based on partial sequences of *16S* rRNA, *18S* rRNA, *28S* rRNA, *cytochrome c oxidase subunit* I (*COI*) and *histone H3* gene markers that indicated the newly identified specimens represent a new genus and species of heteronemertean in the family Lineidae. We also provide morphological data and images illustrating its unique behaviour, contracting its body into a series of rings similar to an accordion. Our results increase our knowledge on the diversity of an important but often overlooked invertebrate phylum and emphasize the need to combine morphological and molecular data to discover new ribbon worm species and better evaluate the true diversity of the phylum.

## 1. Introduction

Ribbon worms are a notoriously difficult group for taxonomic studies. In the past, most species descriptions have been established on the basis of external features, many of which have been proven taxonomically unreliable [1]. To solve this problem, during the last century, the authors started to include morphological characters corresponding to the internal anatomy in species descriptions. Therefore, taxonomic studies of nemerteans typically involved time-consuming histological techniques in which whole specimens were sectioned to describe their entire internal anatomy [2,3]. However, this paradigm has recently been called into question for several reasons, and it is slowly being abandoned in more recent nemertean taxonomic studies [4–7]. Many internal characters are homoplastic or have been overlooked in species descriptions, for example, neurochord cells [4]. In addition, nemerteans are very challenging to identify in preserved samples as live colours are not conserved and external morphological characters are scarce. As a consequence, the *ca* 1350 species currently described [8] might represent just a small fraction of the true diversity of nemerteans [9,10], which is especially worrisome in the face of the current biodiversity crisis. Some studies estimate that up to 1400 additional ribbon worm species remain undescribed [8,11], but the rate of new or cryptic species discovery suggests the actual diversity may be an order of magnitude larger [12–14]. To alleviate these issues and advance our knowledge of nemertean biodiversity at an accelerated pace, Strand & Sundberg [9] proposed to disregard histology and describe new ribbon worm species based on a combination of external characters and DNA sequences. This new system has been adopted by numerous authors in recent taxonomic studies [10,15–17] and allows to fast-track the discovery and description of nemertean diversity and evolution. Here, we also follow this approach to describe a new genus and species of lineid heteronemertean from the northwest coast of Spain.

The number of nemertean species in Spain is relatively low compared with that of other European countries, with only 75 species recorded so far [18]. This probably reflects a lack of taxonomic expertise rather than a true low diversity of the phylum in the region, which is further supported by the fact that nemertean species are frequently reported as *Nemertea* sp. in most benthic studies conducted in Spanish waters [19]. Furthermore, most research on nemertean diversity has been concentrated in just a few regions, Galicia (NW Spain) being the most studied area. As a consequence, the majority of new ribbon worm species described in recent decades have been discovered in this area [18,20,21], giving the false idea that Galician waters are a hot spot of nemertean diversity. One of the advantages of working regularly in a particular area is that you establish bonds of friendship and scientific collaborations with people who are excellent connoisseurs of the underwater fauna due to their work, such as diving instructors. As a result of the sport dives of one of the authors of this paper (J.P.-D.), the occurrence of an unknown ribbon worm was detected in the ría de Arosa (Galicia, Spain). The presence of horizontal lateral cephalic slits allows to place the specimens in the family Lineidae, the most speciose family of the subclass Heteronemertea, with 462 species [8]. Here, based on morphological observations and molecular analyses, we describe these newly discovered specimens as a new genus and species of the phylum Nemertea.

## 2. Material and methods

### 2.1. Sampling and morphological analyses

Specimens of the newly discovered species were collected in the ría de Arosa (Galicia, Spain) by SCUBA diving during the summer of 2021. Specimens were kept alive for imaging and observation of external morphology and then placed in RNA later and stored at −80°C until processed for DNA extraction. One specimen was photographed and preserved in 10% buffered formalin in seawater, subsequently dehydrated in a graded series of ethanol, later transferred to xylene, embedded in 56°C paraffin wax, sectioned at 6 μm, and stained using the Mallory trichrome method for histological examination [22]. Additional specimens sequenced herein and included in the phylogeny were collected in different campaigns around the Spanish coasts, placed in 80% ethanol and stored at 4°C until processed for DNA extraction. Type material and DNA vouchers for the new species are deposited at Museo Nacional de Ciencias Naturales (MNCN) in Madrid (Spain). Catalogue numbers, collection dates, locality and additional relevant information for newly sequenced specimens are listed in electronic supplementary material, table S1.

### 2.2. DNA extraction, amplification and sequencing

Genomic DNA was extracted from a tissue fragment dissected from the posterior end of each specimen with the DNeasy Blood & Tissue Kit (Qiagen), following the manufacturer’s instructions. DNA concentration and integrity were measured in a NanoDrop 8000 (Thermo Fisher Scientific). Fragments of the nuclear genes *18S rRNA* (1589 bp), *28S rRNA* (3469 bp) and *histone H3* (329 bp) and the mitochondrial *16S rRNA* (344 bp) and *cytochrome c oxidase subunit I* (*COI*, 664 bp) were amplified through polymerase chain reaction (PCR). Three overlapping pairs of primers were used to amplify *18S rRNA*, namely 18S1F-18S5R, 18S4F-18S7R and 18Sa2.0−18S9R [23]. Primers 28Sa and 28Srd5b [24] were used to amplify *28S rRNA*. Primers 16SarL and 16SbrH [25] were used to amplify *16S rRNA* and primers jgLCO1490 and jgHCO2198 [26] were used to amplify *COI*. Each PCR reaction consisted of 1.5 μl of DNA template in 13 μl reaction volumes containing 0.5 μl of each 10 mM primer and 12.5 μl of RED Taq DNA Polymerase (VWR, Avantor). PCR amplification and sequencing were carried out largely following methods described by previous authors [12,27]. Using the forward primer of each pair described above, 10 μl of the PCR product was used for sequencing at the Servicio de Secuenciación Sanger, Unidad de Genómica and Universidad Complutense de Madrid and Macrogen. Sequence data were edited in Geneious v. 6.1.6 [28] to remove primers from all sequences and merge the three *18S rRNA* overlapping fragments into a consensus sequence. The multiple sequence alignments for the five different markers were run in the online server of MAFFT v. 7 [29] using the iterative refinement method G-INS-I. Genbank accession numbers for all sequences included in the analysis are listed in the electronic supplementary material, table S2.

### 2.3. Phylogenetic analyses

To evaluate the phylogenetic relationships between the new specimens from Galicia and other Lineidae species, sequences from 5 specimens of the new species were combined with 12 additional specimens newly sequenced here, and with sequences corresponding to 79 species available in GenBank (electronic supplementary material, table S2). A total of 67 species of the family Lineidae were incorporated in the analysis along with 29 species of palaeonemerteans and hoplonemerteans, which were included as outgroups (electronic supplementary material, table S2). We conducted maximum-likelihood (ML) analyses of the concatenated partitioned dataset using both IQTREE v. 1.6.12 [30] and RAxML v. 8.2.12 [31]. For the RAxML analysis, selection of the best model of sequence evolution for each dataset was performed using the Akaike information criterion (AIC) in JModeltest 2 [32]. The best model for each partition was the general time reversible (GTR) with gamma-distributed rates across sites and a proportion of invariable sites (GTR+G + I). The ML analysis was then run in RaxML using the GTR+G + I evolutionary model, and bootstrap (bs) support values were estimated using 1000 replicates and 10 starting trees. We also used IQ-TREE to automatically search for the best-fitting model of sequence evolution and to conduct the ML analysis estimating the best tree and nodal support values simultaneously using the embedded ultrafast bootstrap approach (UFBoot) with 1000 replicates [33] and the SH-like approximate likelihood ratio test (SH-aLRT) also with 1000 replicates [34]. The resulting phylogenetic trees were plotted and edited with iTOL v. 5 [35].

## 3. Results

### 3.1. Taxonomy

PILIDIOPHORA Thollesson and Norenburg, 2003

Class HETERONEMERTEA Coe (1901)

Family LINEIDAE McIntosh, 1874

Genus *Pararosa* gen. nov.

**Diagnosis**. Heteronemertean with a single pair of horizontal lateral cephalic slits which posteriorly form deep intramuscular canals; proboscis simple, unbranched; nervous system without neurochord or neurochord cells; dermis thick, glandular region separated from body wall muscles by well-developed connective tissue layer; blood system with cephalic lacunae; frontal sensory organs consisting of three simple ciliated pits located at the tip of the head; eyes absent.

**Etymology**. The name refers to the type locality of the type species, the ría de Arosa, preceded by the Spanish word par (pair), referring to the two localities where the specimens were collected. The name of the type locality in Spanish is feminine, and thus the new genus name is also feminine.

**Type species**. *Pararosa vigarae* sp. nov.

***Pararosa vigarae*** **sp. nov. Junoy & Verdes**

(Figures 1 and 2)

**Figure 1.**
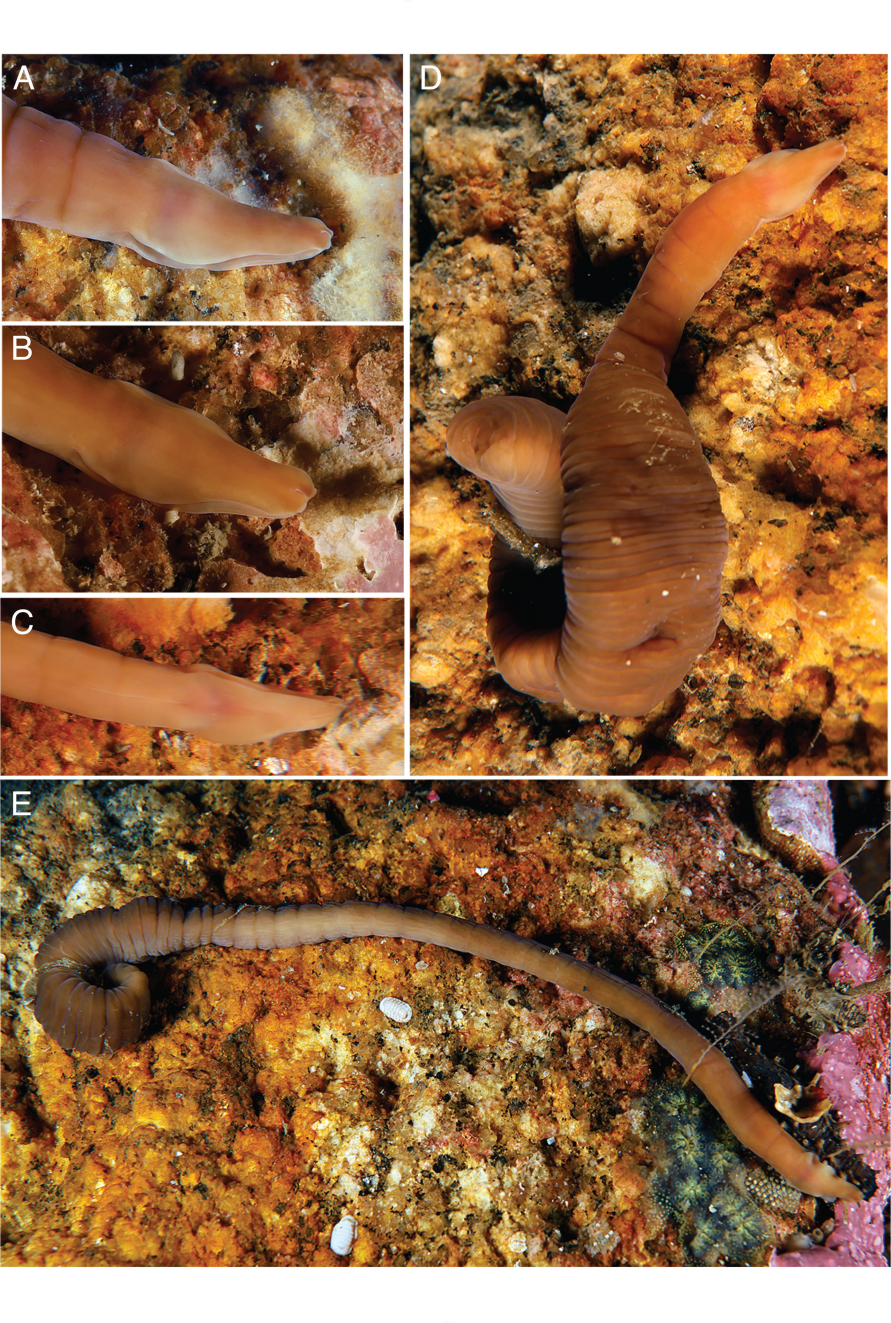
Live images of *P. vigarae* sp. nov. (A) Dorso-lateral view of head, showing cephalic slits; (B) anterior end, dorso-lateral view, showing detail of head tip; (C) ventral view of head, the mouth appears as a whitish middle line just behind the cephalic slits; (D) complete specimen with contracted body, showing epidermal rings; (E) dorsal view of complete specimen in a relaxed state, showing epidermal rings.

**Figure 2.**
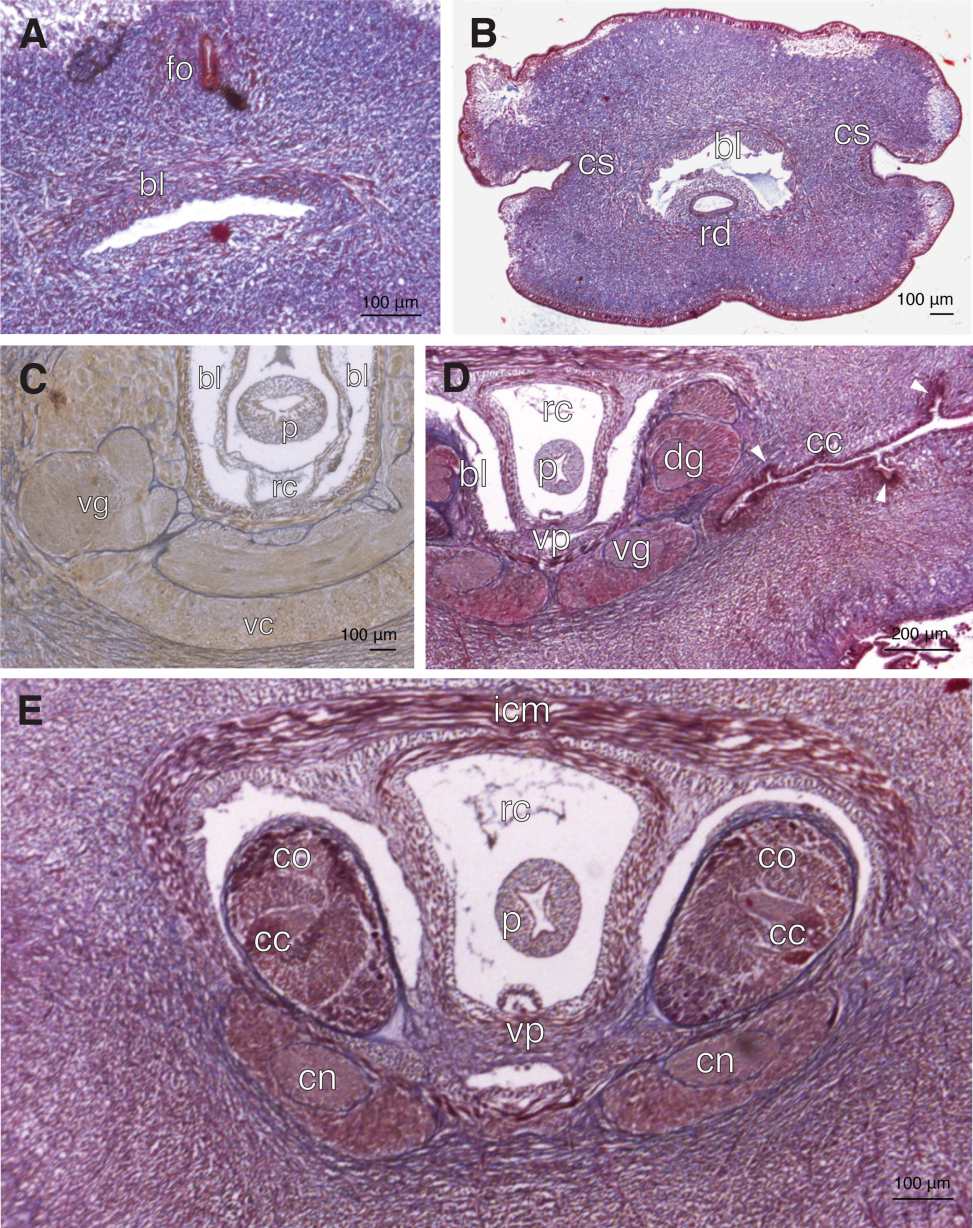
Histological sections of *P. vigarae* sp. nov. (A) Transverse section through head, showing frontal organ and blood lacuna; (B) transverse section through rhynchodaeum, showing cephalic slits; (C) transverse section through ventral cerebral commissure; (D) transverse section through brain, showing ciliated canal pits (arrowheads); (E) transverse section through cerebral organs. Abbreviations: bl, blood lacuna; cc, ciliated cerebral canal; cm, circular muscle layer; co, cerebral organ; cs, cephalic slit; dg, dorsal ganglia; fo, frontal organ; ll, lateral blood lacuna; ln, lateral nerve cord; p, proboscis; rc, rhynchocele rd, rhynchodaeum; rv, rhynchocoelic villus; vc, ventral cerebral commissure; vg, ventral ganglia; vl, ventral blood lacuna.

**Material examined**. Holotype fixed in RNAlater (MNCN-ADN 200.080), four paratypes fixed in RNAlater (MNCN-ADN 200.076−200.079) and one in 96% EtOH (MNCN 5.03/13). Holotype and paratypes fixed in RNAlater collected together under a shell of *Glycymeris glycymeris* [36,36] in a rocky bottom, 32 m depth, A Fanequeira, ría de Arosa (Galicia, Spain), 42°32.3830′ N, 8°57.9860′ W, 23 October 2021. Paratype MNCN 5.03/13 found under a stone in a rocky bottom, 28 m depth, among specimens of the brittle star *Ophiopsila aranea* Forbes, 1843 [37], Os Esqueiros, ría de Arosa (Galicia, Spain), 42°30.5680′ N, 8°56.4500′ W, 17 October 2021.

**Diagnosis**. Heteronemertean with brown to dark green body, head shape retuse; ocelli absent; contracts into regular rings that persist as annular constrictions when stretched.

**Description**. Specimens examined alive were 110−250 mm long and 3−4 mm wide, but are capable of contracting to 1/4 or 1/5 of that length when disturbed. The contraction is rapid and forms regular rings that give it an accordion-like appearance, which we have used to colloquially refer to the species. The number of rings varies according to the size of the specimen, with the largest specimen (25 cm long) presenting 60 rings when fully stretched. These annular epidermal constrictions are frequently observed as darker coloured rings (figure 1A–E). Body coloration is brown to dark green, similar between the dorsal and ventral surfaces. Body width is uniform throughout its length, posterior end tapers to a narrow point (figure 1D). Head shape retuse, apex square with rounded corners, with a dimple in the centre, emarginate, which sometimes disappears to form a small ridge (figure 1B,C). Long, wide and deep pair of horizontal cephalic slits running from the tip of head to the beginning of the mouth; upper and lower margins of the slit do not meet medially, slit open; the truncate posterior margin of the cephalic slits ends just anterior to the mouth (figure 1B,C). No eyes present. Cephalic ganglia are visible as a reddish area just above the mouth (figure 1A).

The internal anatomy of the head shows the typical heteronemertean frontal organ with three ciliated pits, two lateral and one dorsal; however, in the new species, it is observed in the sections open to the exterior into the anterior cephalic notch (figure 2A). The rhynchodaeum is ciliated (figure 2B). The blood system of the head consists of a single lacuna over the rhynchodaeum (figure 2B); just in front of the proboscis insertion the lacuna separates to form a pair of lateral lacunae (figure 2B), the right lacuna penetrates the rhynchocoel lumen originating an elongate rhynchocoelic villus (figure 2C,D). A midventral lacuna is observed under the rhynchocoel. The brain is well developed; both fibrous and ganglionic regions are invested with a thin neurilemma (figure 2C). Neither neurochords, nor neurochord cells are present. The ciliated cerebral canals run inwards from the posterior part of the cephalic slits, showing small pits (figure 2D). These canals lead to large, well-developed cerebral organs (figure 2E). Dermis is well developed, with glands not separated from outer longitudinal muscle layer of body wall by connective tissue stratum.

**Etymology**. Named after Rosa Vigara, wife of the senior author, as a gift for their golden wedding anniversary. Specific epithet is a noun, in reference to the last name Vigara.

**Common name**. Accordion worm. Spanish common name: gusano acordeón.

**Habitat and behaviour**. *Pararosa vigarae* gen. nov. sp. nov. lives under rocks and shells in the subtidal, about 30 m deep. The six specimens were found surrounded by a mucus sheath, five of them sheltered together under a shell of *Glycymeris glycymeris*. In addition to its size and coloration, what distinguishes it from other heteronemertean species is its unique behaviour, as it shrinks into a series of rings, reminiscent of large leeches.

*Bilucernus grubei* (Hubrecht, 1879) comb. nov.

*Lineus grubei* (Hubrecht, 1879)

*Cerebratulus grubei* (Hubrecht, 1879)

**Distribution**. Found in the North Atlantic Ocean and the Mediterranean Sea. The species has been reported in Naples, Italy [38–40]; Madeira, Portugal (41) and Asturias and Ceuta, Spain (18).

**Diagnosis**. The body is coloured dark brown, head with transverse fine white ring, a pair of white spots at the terminal end and two long, horizontal cephalic slits. No boundary between head and trunk, and no caudal cirrus.

**Remarks.** *Bilucernus grubei* closely resembles *B. takakurai*, but in the latter species, the transverse white line is situated slightly further backwards. *Bilucernus grubei* is also similar to *Micrura purpurea* in external appearance, but the latter species has a caudal cirrus, and the tip of the head is yellowish, unlike *B. grubei*, which has a black head.

### 3.2. Phylogenetic analyses and systematics

**Figure 3.**
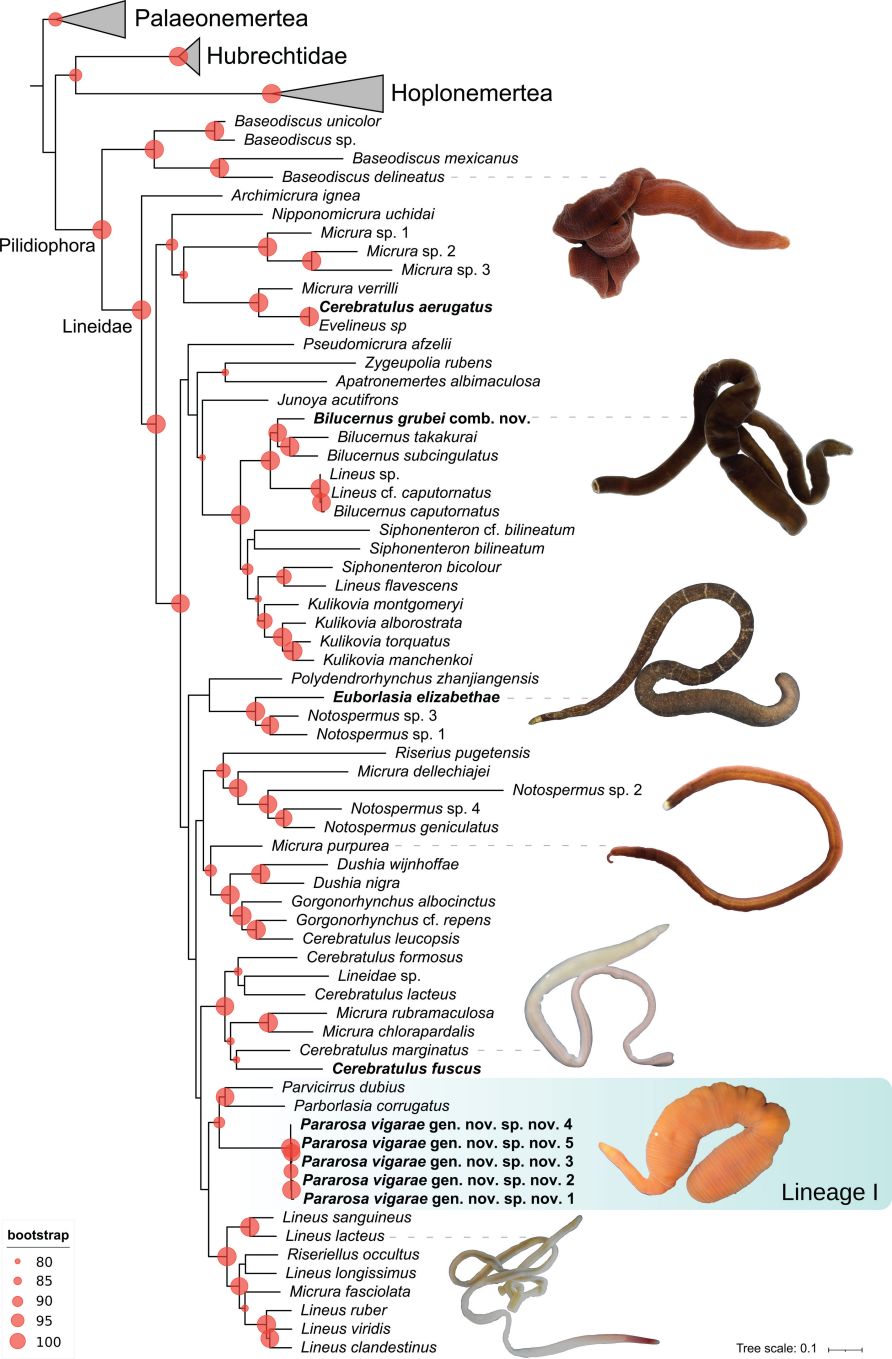
Most probable phylogenetic tree inferred from the ML analysis of a concatenated molecular dataset of partial sequences of 16S rRNA, 18S rRNA, 28S rRNA, cytochrome c oxidase subunit I and histone H3 gene markers. Bootstrap support values (UFBoot) greater than or equal to 80 are indicated with red circles. Lineage I as described by [7] is shaded in blue. Specimens newly sequenced in this study are marked in bold.

To confirm and evaluate the phylogenetic position of the newly discovered genus and species within the family Lineidae and to investigate its evolutionary relationships, we performed a ML phylogenetic analysis with a multilocus alignment comprising 7392 bp generated by concatenating the amplified fragments of *18S rRNA* (2370 bp), *28S rRNA* (3220 bp) and *histone H3* (337 bp) and the mitochondrial *16S rRNA* (624 bp) and *cytochrome c oxidase subunit I* (*COI*, 664 bp). The topology of the phylogenetic trees inferred with the different ML methods (RAxML and IQ-TREE) is almost identical (figure 3; electronic supplementary materials S1 and S2), indicating that our phylogenetic inferences are robust regardless of the tree-building method used. The new specimens are nested within a well-supported clade (bs = 84, UFboot = 88) that includes the species *Parborlasia corrugata* [42] and *Parvicirrus dubius* [43], which corresponds to Lineage I as described by [7] (figure 3; electronic supplementary materials S1 and S2). In addition, our analyses reveal that *L. grubei* [38] s. str. is nested within a well-supported clade (bs = 100, UFboot = 100) that also includes species of the recently established genus *Bilucernus* Ikenaga, Maslakova, Yoshida & Kajihara, 2025 [44], and it is therefore proposed to be renamed as *Bilucernus grubei* comb. nov.

## 4. Discussion

The relatively homogeneous morphology and the homoplasy of characters traditionally employed in heteronemertean identification and classification have led several authors to try to unravel the nemertean Gordian knot using molecular data [7,45]. Using molecular data, a series of well-defined clades representing distinct evolutionary lineages have been recognized, but in most cases, they do not correspond to previously described taxonomic groups based on morphology. This has led to taxonomic reorganizations and the establishment of new taxa such as *Maculaura* Hiebert & Maslakova, 2015 [7,46] or *Kulikovia* Chernyshev, Polyakova, Turanov & Kajihara, 2017 [15]. In the present study, we combine morphological and molecular data to describe a new genus and species of ribbon worm from Spanish waters, *P. vigarae* gen. nov. sp. nov., increasing our knowledge on the diversity of heteronemerteans in the family Lineidae. We provide molecular and morphological information for the new species, as well as molecular data for 12 additional specimens that were also included in the phylogenetic analysis.

*Pararosa vigarae* sp. nov. is nested in a well-supported clade defined as lineid Lineage I by [7] that also includes the species *Parborlasia corrugata* (McIntosh, 1876) [42] and *Parvicirrus dubius* (Verrill, 1879) [43](figure 3; electronic supplementary material, figures S1–S2). These two species are also found as sister taxa in previous molecular studies [16,47], although no shared morphological characters have been identified so far that could represent a synapomorphy for the lineage. *Parborlasia corrugata* is a large species, up to 2 m in length, very common and well known from Antarctic waters [48,49]; whereas *Parvicirrus dubius* is a small species, ranging from 50 to 75 mm in length, distributed in the northwest Atlantic, with striking white eyes and a caudal cirrus [50]. Similar to the new species, *P. corrugata* is capable of contracting its body up to one-fifth of its length [51], but it does not form the characteristic annulations observed in *P. vigarae* sp. nov. (figure 1). These characteristic body constrictions are proposed as an apomorphic character for the new genus and species. When some heteronemertean species contract their body, wrinkles or rings may appear due to epidermal tightening, as observed in *Dushia wijnhoffae* Schwartz & Norenburg, 2019 [52], but in these cases, the rings or constrictions are not regularly distributed as observed in the new species. Moreover, in the new species, these rings persist as epidermal constrictions when the animal is fully stretched. Similar constrictions can be observed in a few species of the nemertean class Hoplonemertea, including *Arenonemertes minutus* Friedrich, 1949 [53], *Annulonemertes minuscula* Berg 1985 [54] and *Nemertellina yamaokai* Kajihara, Gibson & Mawatari, 2000 [55]. According to [56] these external constrictions are not phylogenetically informative and represent convergent structures in these different hoplonemertean species. The characteristic epidermal rings observed in *P. vigarae* gen. nov. sp. nov. would therefore also represent a convergent anatomical feature shared with some hoplonemertean species.

In addition, our analyses revealed that *L. grubei* [38] s. str. is nested with species of the newly established genus *Bilucernus* [44], including *B. caputornatus* (Takakura, 1898) [57], *B. pictifrons* (Coe, 1904) [58], *B. subcingulatus* (Takakura, 1898) and *B. takakurai* Ikenaga, Maslakova, Yoshida & Kajihara, 2025 [44]. As Ikenaga *et al*. [44] suggested based on the external similarity between *L. grubei* s. str. and *B. takakurai*, our phylogenetic analysis confirms that the specimen of *L. grubei* we analysed from Ceuta is in fact a member of *Bilucernus*, and we therefore propose to transfer it to this newly established genus. Our results also show that *Lineus* sp. and *Lineus* cf. *caputornatus* are nested within this clade and therefore, according to their phylogenetic position, these species are probably undescribed congeners of *Bilucernus*. However, the sequences corresponding to these specimens were downloaded from GenBank, and their morphological characteristics could not be examined, therefore, we prefer not to propose a nomenclatural change for these particular species.

In conclusion, the present study increases the number of known ribbon worm species in Spanish waters, with the description of the new genus and species of lineid heteronemertean *P. vigarae* gen. nov. sp. nov., as well as identifying an undescribed species of the newly described genus *Bilucernus* [44]. We provide morphological and molecular information for the *P. vigarae* gen. nov. sp. nov., which is closely related to Lineage I as described by [7] in their proposal for a family-level classification system for lineid heteronemerteans. Our results further emphasize the need to incorporate a combination of morphological and molecular data to discover and describe new ribbon worm species, in order to better evaluate the true diversity of the phylum.

## Data Availability

Genbank accession numbers for all DNA sequences used in this study are listed in electronic supplementary material, table S2 [59].
